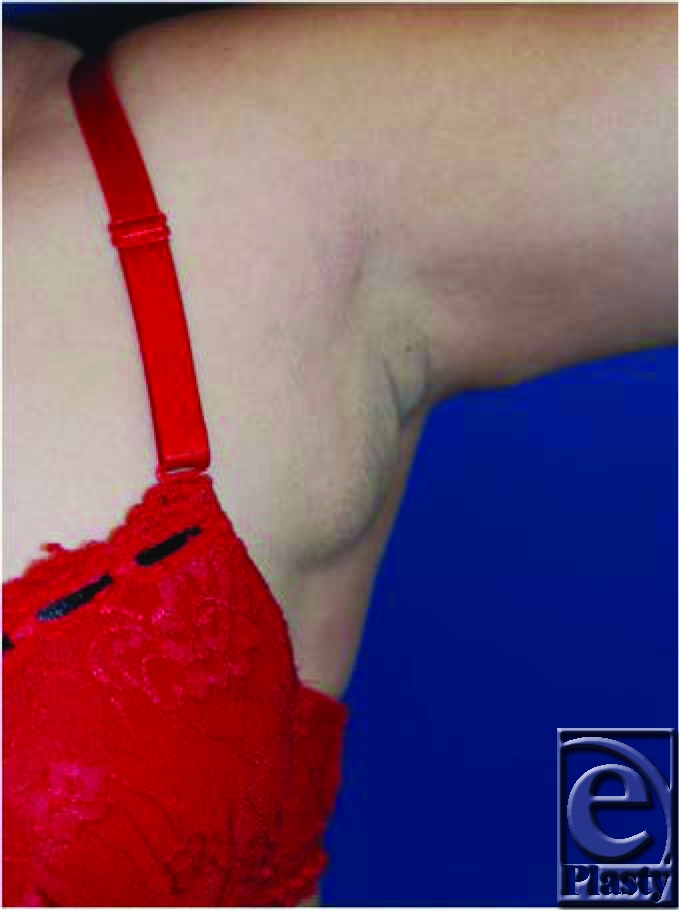# Accessory Breast Tissue

**Published:** 2012-04-23

**Authors:** Priti P. Patel, Ahmed M. S. Ibrahim, Jacob Zhang, John T. Nguyen, Samuel J. Lin, Bernard T. Lee

**Affiliations:** Beth Israel Deaconess Medical Center/Harvard Medical School, Brookline Avenue, Boston, MA

## DESCRIPTION

A 36-year-old woman presents with significant pain and swelling over the left axillary area, which she reports worsened over the last 3 years. The tenderness becomes more prominent with her menstrual cycle. The patient denies any other changes in her breast.

## QUESTIONS

1. **What is the most likely pathology in this scenario?**
2. **What is the best course of treatment for this patient?**
3. **Discuss the classification system for this condition.**

## DISCUSSION

Polymastia (supernumerary breasts) is a relatively common congenital condition in which abnormal accessory breast tissue is found in addition to normal breast tissue. However, it may not be evident until puberty.^1^^,^^2^ Although accessory breast tissue is usually found along the thoracoabdominal region of the milk line (67%), which extends down to the groin, ectopic breast tissue may also be present in locations such as the face, back, and thigh.^1^^,^^3^^,^^4^

About 2% to 6% of females and 1% to 3% of males are affected by this condition, a third of whom have more than one area of supernumerary tissue growth. Occurrence rates vary widely on the basis of ethnicity and gender, ranging from as low as 0.6% in Caucasians to as high as 5% in Japanese females.^3^^,^^4^

Symptoms include swelling and tenderness of the affected region, thickening of the axilla and limited range of shoulder motion, and irritation from clothing. These symptoms are usually worsened by the onset of puberty and pregnancy. Supernumerary breast tissue also develops during this period alongside normal breast tissue growth.^1^

Polymastia was categorized in 1915 by Kajava, whose classification system still remains in use today.^3^^-^5

Class I consists of a complete breast including glandular tissue, nipple, and areola.

Class II consists of only glandular tissue and nipple, without areola.

Class III consists of only glandular tissue and areola, without nipple.

Class IV consists of only glandular tissue.

Class V (pseudomamma) consists of only nipple and areola, without glandular tissue.

Class VI (polythelia) consists of only the nipple.

Class VII (polythelia areolaris) consists of only the areola.

Class VIII (polythelia pilosa) consists of only hair.

The development of breast neoplasm in ectopically located glandular tissue has been depicted. Madej et al^6^ describe a rare case of a 50-year-old woman who, in spite of undergoing regular mammography screening, developed an invasive accessory breast cancer. On physical examination, a 2-cm tumor located 4 cm below the left inframammary fold was detected, which infiltrated the thoracic wall. Biopsy revealed an invasive stage IIIB ductal breast cancer. The patient was managed by chemotherapy and surgical excision. The authors advocate that a diagnosis of accessory breast cancer be made by physical examination and ultrasonography. When a diagnosis of symptomatic accessory breast is made, preventive resection of accessory breast may be the treatment of choice.

There are many possible treatments to correct polymastia; however, it is generally recommended that accessory breast tissue be excised.^1^^,^^7^ Sadove and van Aalst^8^ reported that when compared to other congenital and acquired breast anomalies, correction of polymastia with breast reduction techniques required the least number of operations per patient. To assess the surgical treatment of axillary accessory breast tissue, Aydogan et al^9^ performed a retrospective analysis of 29 patients over a period of 8 years, of which 16 patients had unilateral and 13 patients had bilateral accessory breasts; 21 patients underwent excision of breast tissue, 5 had liposuction, and 3 had both. It was reported that excision, liposuction, or both resulted in satisfactory outcomes. Fan^10^ describes an alternative tumescent liposuction technique to prevent the occurrence of the central depression appearance that is often left as a result of the adjacent fat tissue remnant following the traditional methods of resecting the accessory breast tissue. This alternative approach may result in minimal scarring and better contour than can be obtained by conventional methods.

Regardless of the technique utilized, attempts at removing accessory breast tissue can lead to surgical complications such as contour irregularities, seromas, and possibly recurrence. Large resections often benefit from postoperative drain usage.

## Figures and Tables

**Figure:**